# Copper (II) complexes of bidentate ligands exhibit potent anti-cancer activity regardless of platinum sensitivity status

**DOI:** 10.1007/s10637-017-0488-2

**Published:** 2017-07-21

**Authors:** Mohamed Wehbe, Cody Lo, Ada W. Y. Leung, Wieslawa H. Dragowska, Gemma M. Ryan, Marcel B. Bally

**Affiliations:** 1 Experimental Therapeutics, British Columbia Cancer Agency, 675 West 10th Avenue, Vancouver, BC V5Z 1L3 Canada; 2 Faculty of Pharmaceutical Sciences, University of British Columbia, 2146 East Mall, Vancouver, BC V6T 1Z3 Canada; 3 Department of Pathology and Laboratory Medicine, University of British Columbia, 2211 Wesbrook Mall, Vancouver, BC V6T 2B5 Canada; 4 Center for Drug Research and Development, Vancouver, BC V6T 1Z4 Canada

**Keywords:** Cisplatin, Platinum drugs, platinum resistant cancer, Diethyldithiocarbamate, Plumbagin, Pyrithione, 8-hydroxyquinoline, Clioquinol, Carboplatin, Copper complexes, Copper based therapeutics

## Abstract

Insensitivity to platinum, either through inherent or acquired resistance, is a major clinical problem in the treatment of many solid tumors. Here, we explored the therapeutic potential of diethyldithiocarbamate (DDC), pyrithione (Pyr), plumbagin (Plum), 8-hydroxyquinoline (8-HQ), clioquinol (CQ) copper complexes in a panel of cancer cell lines that differ in their sensitivity to platins (cisplatin/carboplatin) using a high-content imaging system. Our data suggest that the copper complexes were effective against both platinum sensitive (IC_50_ ~ 1 μM platinum) and insensitive (IC_50_ > 5 μM platinum) cell lines. Furthermore, copper complexes of DDC, Pyr and 8-HQ had greater therapeutic activity compared to the copper-free ligands in all cell lines; whereas the copper-dependent activities of Plum and CQ were cell-line specific. Four of the copper complexes (Cu(DDC)_2_, Cu(Pyr)_2_, Cu(Plum)_2_ and Cu(8-HQ)_2_) showed IC_50_ values less than that of cisplatin in all tested cell lines. The complex copper DDC (Cu(DDC)_2_) was selected for in vivo evaluation due to its low nano-molar range activity in vitro and the availability of an injectable liposomal formulation. Liposomal (Cu(DDC)_2_) was tested in a fast-growing platinum-resistant A2780-CP ovarian xenograft model and was found to achieve a statistically significant reduction (50%; *p* < 0.05) in tumour size. This work supports the potential use of copper-based therapeutics to treat cancers that are insensitive to platinum drugs.

## Introduction

Platinum (Pt) drugs are the most successful class of inorganic medicinal compounds used to treat cancer [1, 2]. They are a mainstay in cancer therapy, being utilized in approximately 50% of chemotherapeutic regimens [2, 3]. Cisplatin (CDDP) was first used to treat leukemia in the 1960s, but through several inorganic medicinal chemistry programs, other Pt-based drugs (Carboplatin (CBDCA), Oxaliplatin, Paraplatin etc.) have been produced and approved by the FDA [2, 4]. Mechanistically, these drugs are known to act by forming Pt-DNA complexes that cause DNA damage that accumulate to a point that is beyond repair, ultimately leading to cell death [3, 5]. Pt drugs are currently used as first-line therapy in blood, lung, ovarian, testicular, and head and neck cancers. While some cancers are Pt sensitive and thus respond to Pt drugs [2], many others are Pt-insensitive due to inherent or acquired resistance [3, 5]. There is a need to define drugs capable of treating Pt insensitive cancers [6].

While Pt drugs have been successful for many patients, they also produce serious side effects including nephrotoxicity, neurotoxicity and ototoxicity [7]. To address these adverse effects, many inorganic medicinal chemistry investigations have focused on developing alternatives to Pt-based drugs by replacing Pt with other divalent metals, such as copper [8]. Copper-based therapeutics are generally less toxic owing to the physiological processes that detoxifying excess of copper [9]. In line with this approach, many groups have demonstrated in vitro that copper complexes of natural compounds have anticancer properties; some examples include dithiocarbamates and analogues of 8-hydroxyquinoline [10, 11]. To date, however, no copper complex has transitioned into clinical use for any indication in humans. While the platins that are used in the clinic are water soluble, one major challenge of copper-based therapeutics is their inherently poor aqueous solubility [12]. This problem has made in vivo testing very challenging and required solvents containing mixtures of DMSO and Cremphor which are not clinic applicable due their high toxicity [13, 14]. Recently, we solved the solubility issue of copper-based therapeutics by synthesising copper complexes within liposomes [12]. This technology, referred herein as Metaplex™, was used to examine the effects of copper DDC (Cu(DDC)_2_) in animals bearing a xenograft tumours [15].

**Scheme 1:**
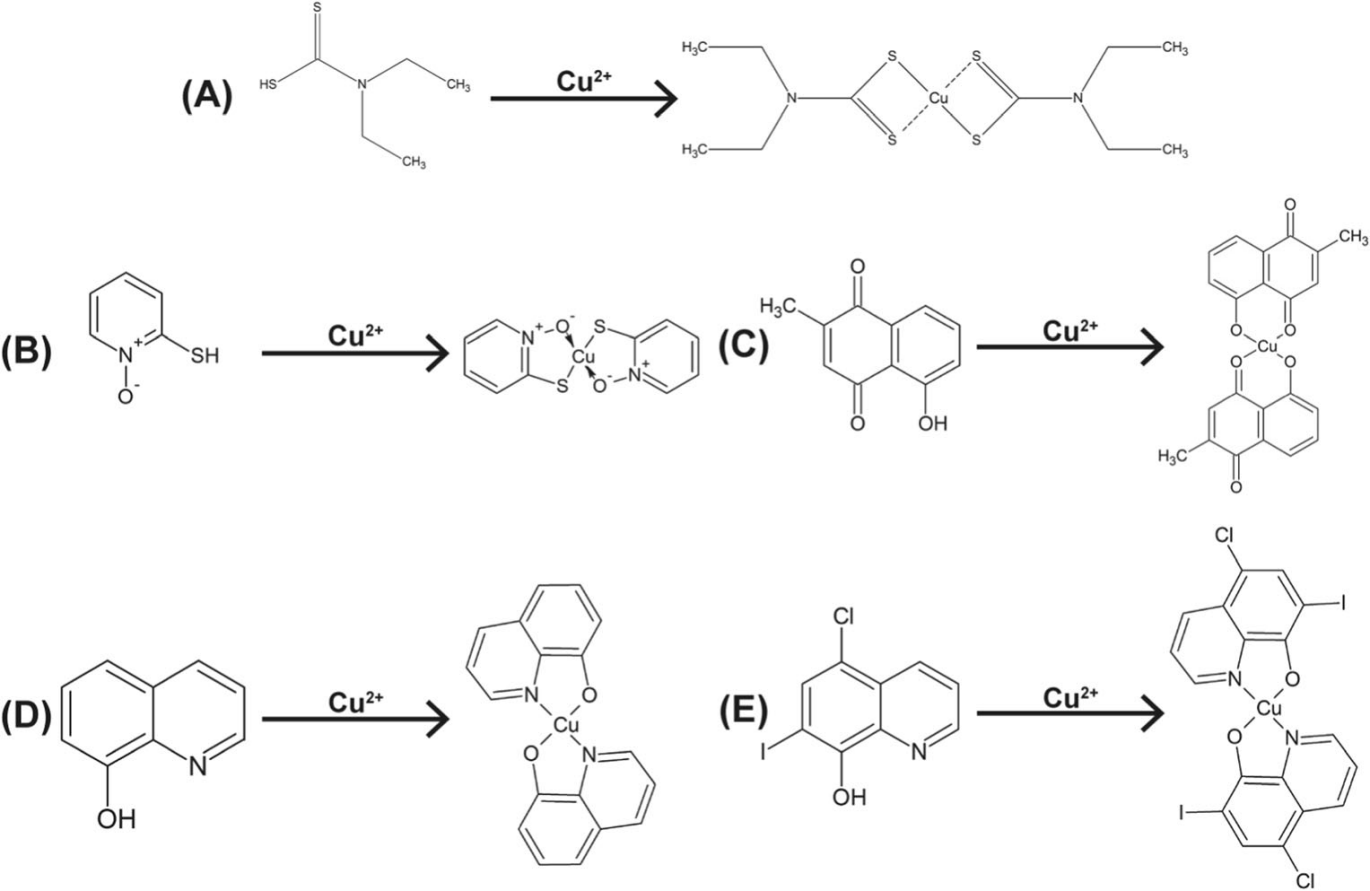
Ligands and their respective copper complex structures. **a** DDC and Cu(DDC)_2_ (**b**) Pyr and Cu(Pyr)_2_. (**c**)Plum and Cu(Plum)_2_. (**d**) 8-HQ and Cu(8-HQ)_2_ and (**e**) CQ and Cu(CQ)_2_

**Scheme 2:**
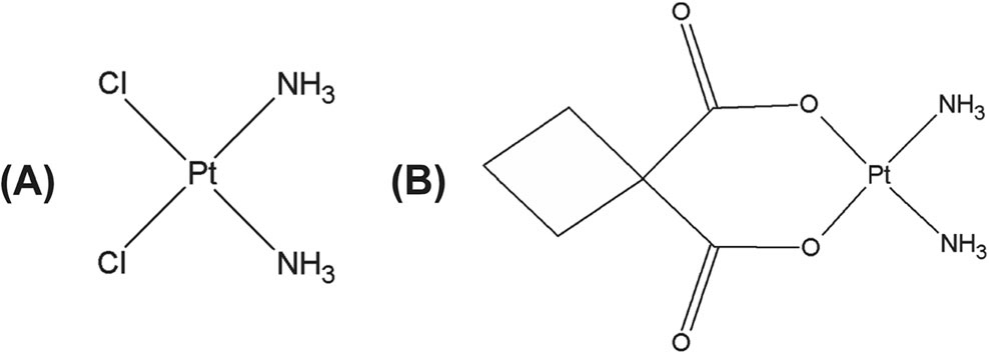
Structures of commonly used platinum drugs. **a** Cisplatin and (**b**) Carboplatin

In these studies, we selected five bidentate copper ligands (Scheme 1) and screened both the ligand and the corresponding copper complex in a panel of eight cancer cells. As indicated above diethyldithiocarbamate (DDC) [16, 17], Pyrithione (Pyr) [13], Plumbagin (Plum) [18, 19], 8-hydroquinoline (8-HQ) [17, 20, 21] and Clioquinol (CQ) [10, 17, 22, 23] have all been shown to have anticancer activity but have never been tested directly against Pt-resistant cancers. Here we compare the activity of these complexes to the activity of CDDP and CBDCA (Scheme 2). The screen showed that many copper complexes are more active in vitro than the Pt controls. We also demonstrate that a Metaplex™ formulation of copper DDC (Cu(DDC)_2_) was active in animals bearing Pt-resistant A2780-CP xenograft tumours. The results highlight the potential of the copper-based therapeutics as candidates to treat Pt resistant cancers.

## Materials and methods

### Materials

Plumbagin was obtained from *Plumbago indica*. Pyrithione (2-Mercaptopyridine N-oxide sodium salt), 8-Hydroxyquinoline, Sodium Diethyldithiocarbamate trihydrate, Copper Sulfate, Clioquinol (5-Chloro-7-iodo-8-quinolinol), and all other chemicals were purchased from Sigma Aldrich. Carboplatin (CBDCA) and Cisplatin (CDDP) were obtained from Hospira. 1,2-distearoyl-sn-glycero-3-phosphocholine (DSPC) and Cholesterol (Chol) were purchased from Avanti Polar Lipids (Alabaster, AL).

### Cell lines

The A549, FaDu, Cal-27, SCC-25, and MV-4-11 cell lines were purchased from ATCC. A2780-S and A2780-CP cell lines were obtained from Dr. Mark W. Nachtigal at the University of Manitoba (Winnipeg, Canada) and the H1933 cells were provided by Dr. William Lockwood at the BC Cancer Agency’s Vancouver Research Centre (Vancouver, Canada). All cell lines were used for up to eighteen passages. A549 and H1933 cells were maintained in RPMI (Gibco). A2780-S, A2780-CP and SCC-25 were maintained in DMEM/F12 (Gibco). MV-4-11, Cal-27, and FaDu cells were maintained in IMDM (Gibco), DMEM (Gibco), and MEM (Gibco), respectively. Media for all cell lines was supplemented with 2 mM L-glutamine (Gibco) and 10% fetal bovine serum (Gibco) and maintained at 37 °C and 5% CO_2_. Media for the SCC-25 cell line was also supplemented with 400 ng/mL hydrocortisone. All cell lines tested negative for mycoplasma when obtained by the lab. Prior to drug treatment, the cells were seeded into 384 well plates and incubated 24 h in media prior to treatment.

### Cytotoxicity assay of Pt drugs, ligands and copper complexes

For in vitro testing, copper complexes were synthesized prior to cell treatment by mixing CuSO_4_ and the ligand at a fixed 2:1 ratio in DMSO (50%). The ligands DDC, CQ, 8-HQ, Plum were solubilised in DMSO and Pyr was dissolved in sterile water. CBDCA and CDDP were dissolved in sterile saline (0.9%).

The adherent cell lines (A549, A2780-S, A2780-CP, FaDu, Cal-27, SCC-25, H1933) were exposed to the indicated compounds in triplicate wells for 72 h. Following treatment, cells were stained in situ with Hoechst 33,342 and ethidium homodimer-I to differentiate between viable (Hoechst-positive/ethidium homodimer-negative) and dead cells that had lost membrane integrity (Hoechst-positive/ethidium-positive). Cells were imaged with the IN Cell Analyzer 2200 (GE Healthcare Life Sciences) and 4 images/well were collected. Images were analyzed with the ToolBox Developer 1.9 software (GE Healthcare Life Sciences) to obtain viable and dead cell counts based on differential staining of cell nuclei. The suspension cell line (MV-4-11) was treated for 72 h and viability was assessed using the Presto Blue™ assay (Life Technologies) following the manufacturer’s instructions. The viability data were normalized to vehicle control (0.5% DMSO in media) and expressed as fraction affected where value of 1 corresponded to 100% loss of cell viability relative to vehicle controls and 0 corresponds to a viability comparable to control cells in culture.

### Cu(DDC)_2_ liposome preparation

For efficacy studies, copper complexes were synthesized inside liposomes, as previously described [12]. Liposomes were prepared using the extrusion method which has been well documented by others [24]. In brief, lipids were removed from freezer and desiccated for 2 h prior to being weighed and dissolved in chloroform at a mol:mol ratio of 55:45 (DSPC:Chol). A non-exchangeable and non-metabolizable lipid marker, ^3^H- cholesteryl hexadecyl ether, was incorporated into the chloroform mixture and the solution was dried of chloroform using nitrogen gas and a thin film was generated using a high vacuum for 2 h. The lipid film was rehydrated using 300 mM CuSO_4_ and extruded for at least 10 passes through a high pressure LIPEX extruder (Evonik Transferra Nanosciences, BC, Canada) using 0.1 μm polycarbonate filters. The final liposome size was determined by quasi-electric light scattering with a ZetaPALS analyzer (Brookhaven). The unencapsulated copper was removed via size exclusion chromatography (SEC; Sephadex G50) and replaced with a buffer containing sucrose (300 mM), HEPES (20 mM) and EDTA (15 mM) (SHE buffer, pH 7.4). The resulting copper-free liposomes were dialyzed against a sucrose (300 mM) and HEPES (20 mM) buffer (SH buffer, pH 7.4) and used for Cu(DDC)_2_ synthesis within the liposomes.

Cu(DDC)_2_ synthesis inside the liposomes was performed at 25 °C in a round bottom flask. Copper-containing liposomes (20 mM) were mixed with DDC (10 mM) for 1 h. As DDC permeates across the liposomal lipid bilayer it interacts with the encapsulated copper to form Cu(DDC)_2._ The free, un-reacted, DDC was removed by SEC and the Cu(DDC)_2_ containing liposomes, collected in the excluded volume were used for in vivo tumour efficacy studies.

### In vivo efficacy study

A2780-CP cells that were used for subcutaneous (sc) implantation were between passages 3–10 and maintained in DMEM/F12 (Gibco) at a confluence of 80–90%. NRG mice (7 per group) were inoculated sc with 1 × 10^6^ cells in a volume of 50 μL using a 28-gauge needle. Treatment was initiated on day four and treatment groups included the vehicle control (SH buffer), CuSO_4_-liposomes (1.7 mg/kg copper, 50 mg/kg lipid), and Cu(DDC)_2_ liposomes (8 mg/kg Cu(DDC)_2_ and 50 mg/kg lipid) on a Monday, Wednesday and Friday ×2 weeks dosing schedule. The copper dose of the CuSO_4_ liposome control was equivalent to that of Cu(DDC)_2_.

Animals were monitored at least three times weekly for body weight and tumour growth which was measured with calipers and tumour volumes were calculated based on a formula of (tumour length x tumour width^2^)/2. Animals were terminated by isoflorane followed by CO_2_ asphyxiation when animals reached a humane endpoint for these studies was defined when tumours exceeded 800 mm^3^ or when tumours ulcerated.

### Statistical analysis

All data were plotted as mean ± SEM or mean ± SD, as described in the figure legends. The IC_50_ values and 95% confidence intervals (CI) were extrapolated from nonlinear regression (curve fit) of the cytotoxicity curves using Prism 6.0 (GraphPad software). To determine whether the cytotoxic effects of copper complexes are associated with platinum sensitivity, the IC_50_ values of each copper complex was plotted against the CDDP IC_50_ for each cell line. Each data point represents one cell line. The Pearson Correlation coefficient and corresponding two-tail *p*-values were then determined using Prism 6.0 (GraphPad software). Tumour volumes between different treatment groups were compared using one-way ANOVA followed by Tukey adjustments to correct for multiple comparisons using Prism 6.0. An adjusted *P*-value <0.05 was considered statistically significant.

## Results

### Pt-resistance does not impact sensitivity of cancer cells to DDC copper complexes

**Fig. 1:**
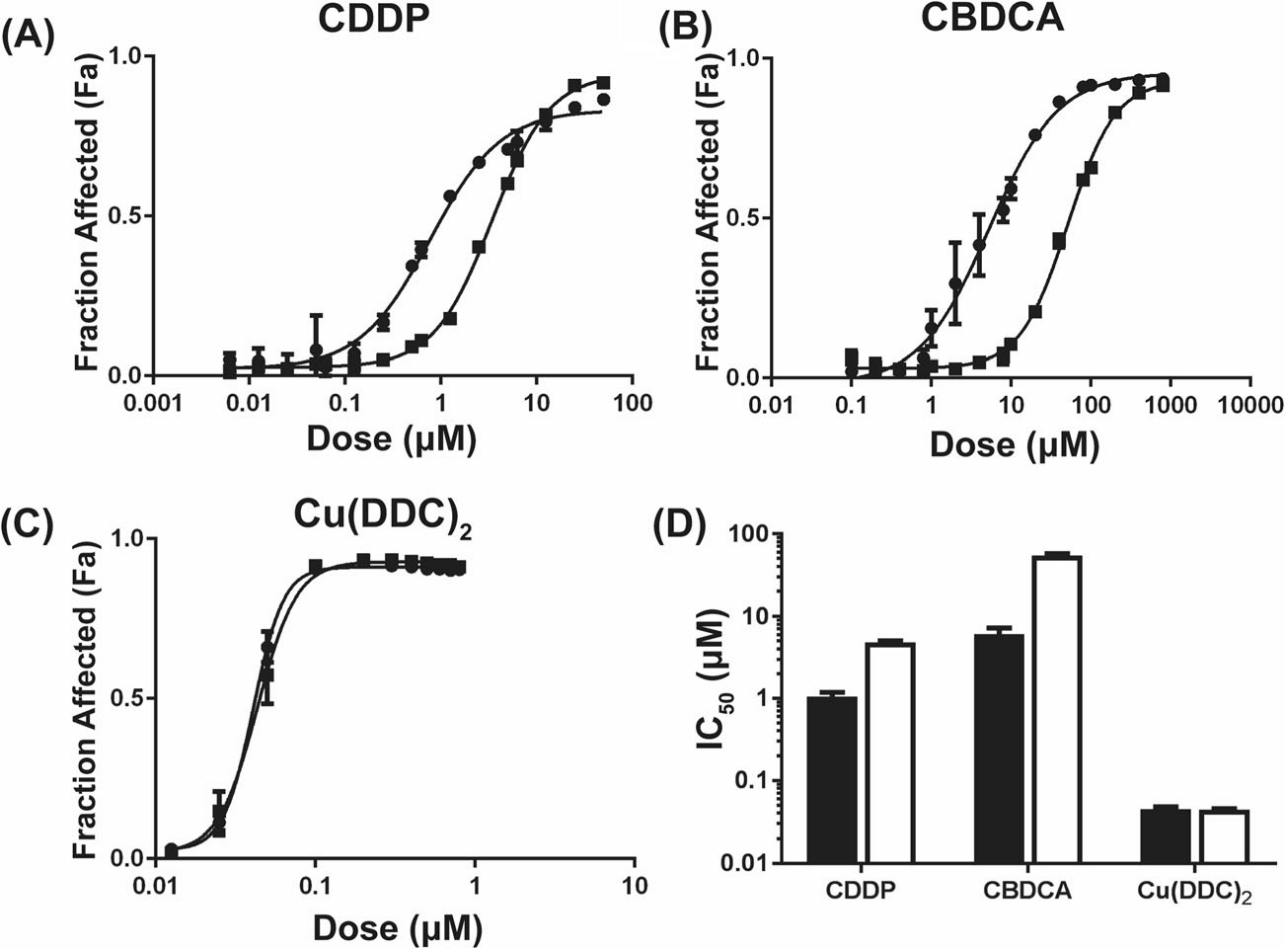
Cytotoxicity profiles of Pt sensitive A2780-S (●) and resistant A2780-CP (■) ovarian cancer cells (or) following 72 h treatment with (**a**) CDDP, (**b**) CBDCA and (**c**) Cu(DDC)_2_. Fraction affected cells was assessed based on viability data normalized to vehicle controls. (**d**) The IC_50_ ± 95% CI for A2780-S *(black*) and A2780-CP (*white*) after treatment with CDDP, CBDCA and Cu(DDC)_2_. Data is presented as the mean of 3 independent experiments ± SEM

CDDP and CBDCA, two commonly used Pt-based therapeutics were tested in a pair of isogenic ovarian cancer cells: A2780-S cells are the parental cells that are sensitive to CDDP while A2780-CP cells are platinum-resistant. As shown in Fig. 1a and b, the IC_50_ of CDDP and CBDCA was 3.7-and 8.5-fold greater, respectively, in the Pt resistant cells when compared to Pt sensitive cells. To determine if Pt resistance impacts activity of copper complexes, A2780-S and A2780-CP cells were treated with Cu(DDC)_2_. The data in Fig. 1c show that the IC_50_ value of the copper complex was not different for Pt sensitive or resistant cells. These data suggest that copper complexes may be effective regardless of Pt sensitivity status.

### Activity of copper complexes in cancer cells with different Pt sensitivity

**Fig. 2:**
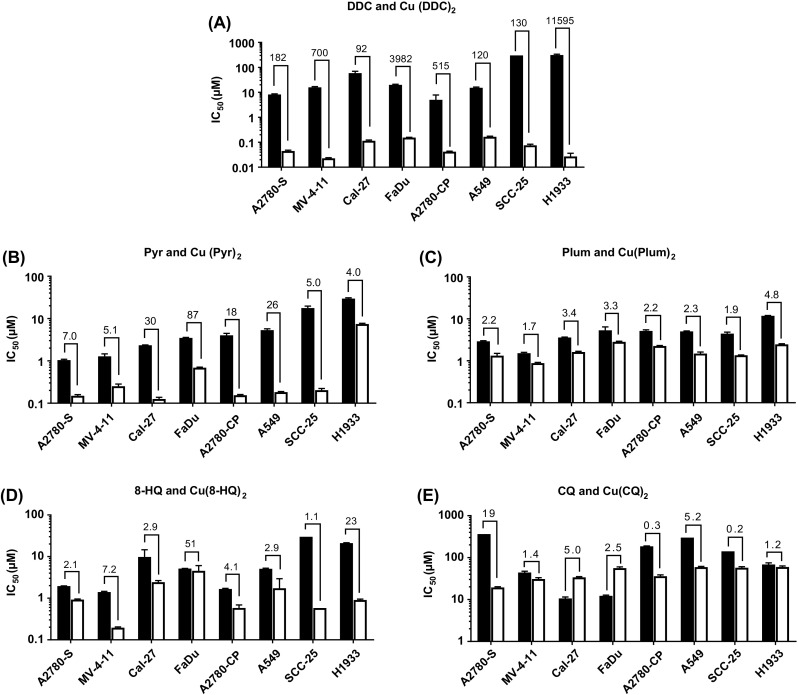
Ligand/drug and copper complex cytotoxicity. A panel of 8 cancer cell lines (A2780-S, MV-4-11, A549, SCC-25, Cal-27, A2780-CP, FaDu and H1933) were treated for 72 h with the ligand/drug (■) or copper complex (□): (**a**) DDC/Cu(DDC)_2_, (**b**) Pyr/Cu(Pyr)_2_,(**c**) Plum/Cu(Plum)_2_, (**d**) 8-HQ/Cu(8-HQ) _2_ and (**e**) CQ/Cu(CQ) _2_. The IC_50_ values were obtained based on the viability data acquired with IN Cell 2200 or PrestoBlue® (M-V-411). The fold difference between the IC_50_ of the ligand and respective copper complex is shown above the histogram bars

The IC_50_ values ±95% confidence intervals for the ligands and their corresponding copper complexes are shown in Fig. 2. Cells were treated with the ligand/drug or complex (2:1 ligand:Cu) for 72 h. All cell lines were also exposed to CuSO_4_ as a control to ensure that copper alone did not impact viability (IC_50_ > 10 μM for all cell lines, data not shown). The results in Fig. 2a indicate that treatment of different cells with the ligand DDC results in an IC_50_ ranging from 5 to 300 μM DDC. In contrast, the IC_50_ for the Cu(DDC)_2_ ranged from 0.02–0.15 μM corresponding to differences of 90–11,000 fold (Fig. 2a). The IC_50_ of Pyr ranged from 1 to 30 μM, and when used as a copper complex (Cu(Pyr)_2_), the IC_50_ decreased to 0.1–7.4 μM (Fig. 2b). The IC_50_ of Plum ranged from 1.5–11 μM while the IC_50_ of and Cu(Plum)_2_ Ranged from 0.8–3 μM in the tested cell lines. In some cell lines (A2780-S, A2780-CP, MV-4-11, Cal-27, FaDu) there was only approximately a 2-fold difference in activity between Plum and Cu(Plum)_2_ (Fig. 2c). Considering the 2:1 (ligand:Cu) complexation ratio, these data indicate that the copper Plum complex is as active as the uncomplexed ligand, suggesting that the cytotoxic effects observed for Plum is copper-independent. 8-HQ was more active as a copper complex (IC_50_ for Cu(8-HQ)_2_ ranged between 0.2–4.5 μM compared to 1.5-30 μM for 8-HQ); however, in A2780-S and FaDu cells, the toxicity of 8-HQ appeared to be copper independent (Fig. 2d). The IC_50_ for CQ and Cu(CQ)_2_ ranged from 10 to 350 μM and 20-60 μM, respectively. Here the dependence on copper complexation for CQ cytotoxicity appears to be cell line-specific: copper-dependent activity was observed in A2780-S, A2780-CP and A549 cells, whereas in MV-4-11, SCC-25 and H1933, the cytotoxicity of CQ was copper-independent. Moreover, CQ appears to be more cytotoxic than Cu(CQ)_2_ in Cal-27 and FaDu cells (Fig. 2e).

**Fig. 3:**
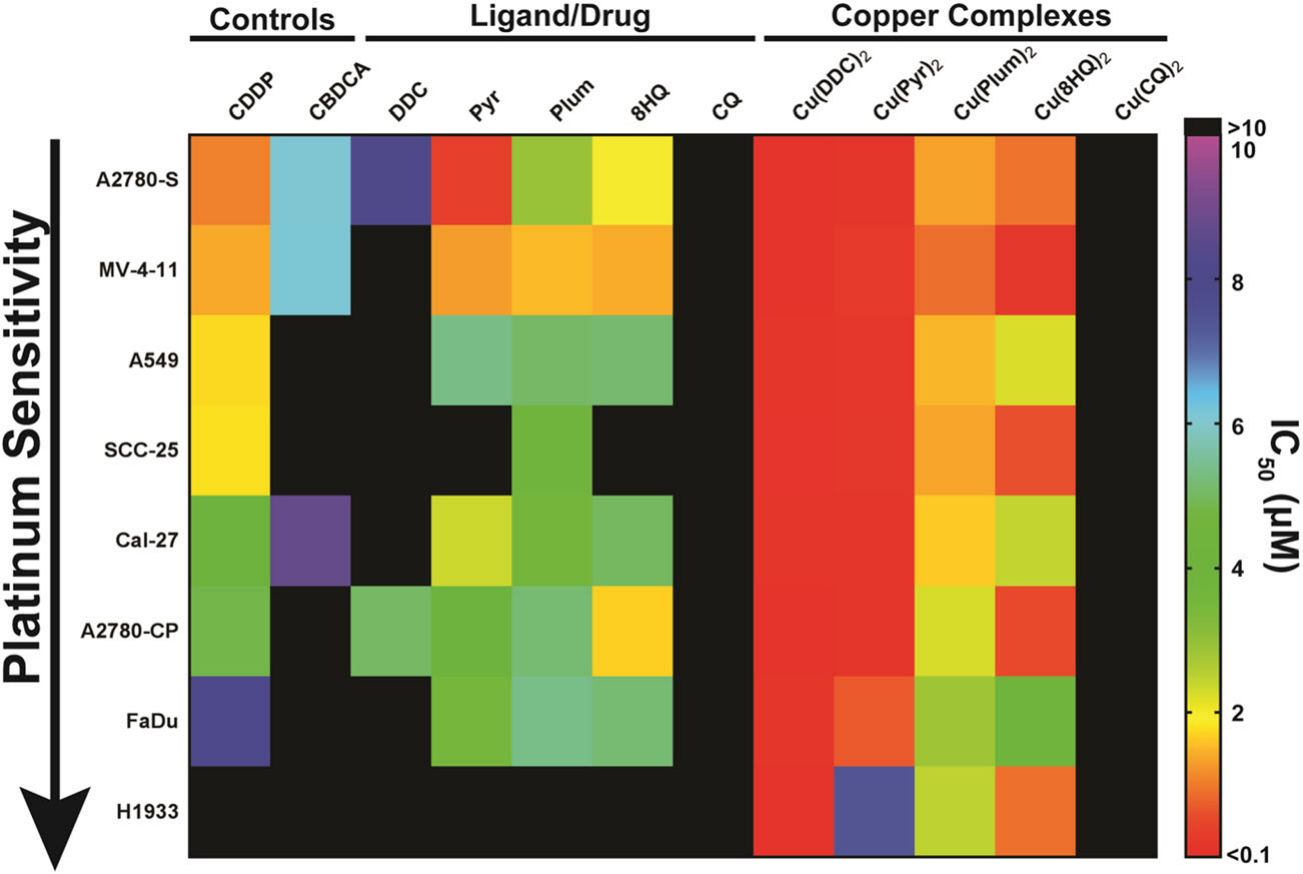
Screen of copper based therapeutics in cancer cells with different Pt sensitivities. IC_50_ values for Pt drugs (CDDP and CBDCA), ligands (DDC, Pyr, Plum, 8-HQ, CQ) and respective copper complexes are shown for cancer cell lines of differing origin arranged in order of sensitivity to CDDP. IC_50_ values were calculated from viability data (*n* = 3 experiments/each cell line) obtained with IN Cell Analyzer 2200 platform or PrestoBlue™ assay (MV-4-11 cells). The agents with IC_50_ values >10 uM (*black*) are considered inactive

The IC_50_s obtained from cytotoxicity assays for each cell line were arranged as a heat-map in order of Pt sensitivity to CDDP (Fig. 3). An IC_50_ cut-off of 10 μM was used to distinguish more potent agents from those that were regarded as pharmaceutically inactive. In general, the ligands appear to be less active in the Pt resistant cell lines whereas the copper complexes augmented anti-cancer activity considerably. The Pyr, Plum and 8-HQ ligands showed activity in most cell lines, however, their activity was enhanced in the tested cell lines when used as complexes with copper; where the greatest increases were observed for Cu(Pyr)_2_. DDC and CQ were the least active ligands. The Cu(CQ)_2_ complex did not show improvement in cytotoxicity whereas the copper complex of DDC was the most active in all cell lines. In general, except for Cu(Pyr)_2_, the activity of the copper complexes used in our study show no correlation with Pt CDDP sensitivity in tested cell lines (Table 1). The heatmap indicates that (Cu(DDC)_2_ is the most effective complex tested against Pt-resistant cancers.

**Table 1:** Correlation between CDDP and copper complex sensitivity based on the IC_50_ values

Drug	Cu(DDC)_2_	Cu(Pyr)_2_	Cu(Plum)_2_	Cu(8-HQ)_2_	Cu(CQ)_2_
Pearson R^2^	0.0680	0.6966	0.1030	0.0880	0.4365
*P*-value	0.0.5325	0.0100	0.4382	0.4755	0.0745
Significant	No	Yes	No	No	No

### Efficacy of liposomal cu(DDC)_2_ in a Pt-resistant A2780-CP tumour xenograft model

**Fig. 4:**
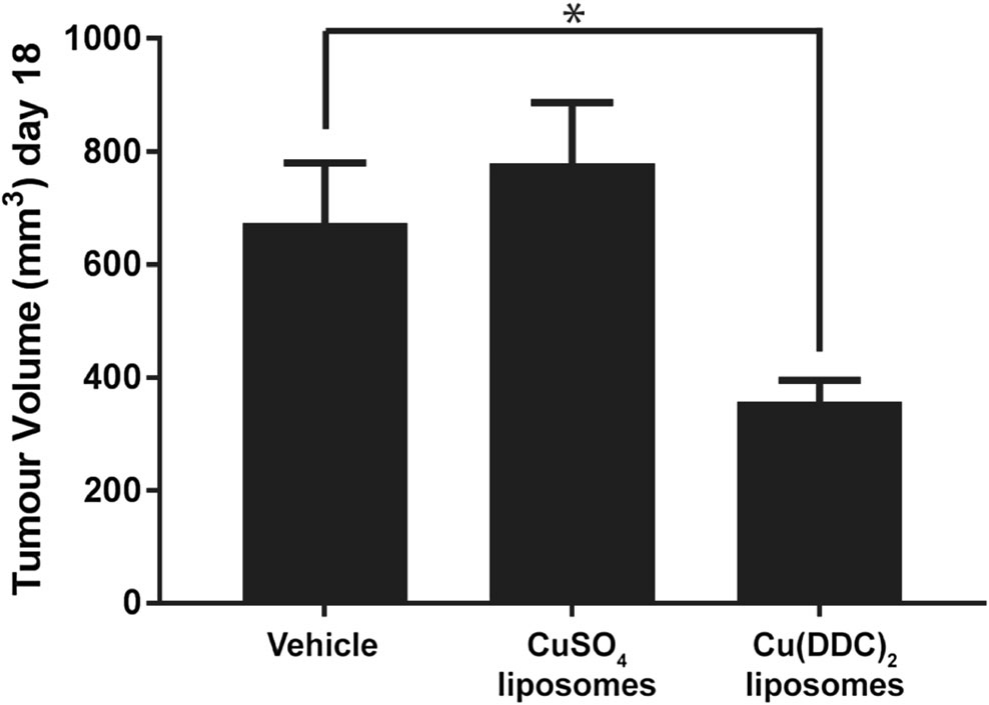
In vivo activity of liposomal Cu(DDC)_2_ in animals bearing Pt resistant A2780-CP tumours. 1 × 10^6^ A2780-CP cells were inoculated sc into immune-compromised NRG mice which were subsequently treated intravenously with vehicle (SH buffer), CuSO_4_-liposomes (1.7 mg/kg copper, 50 mg/kg lipid), and Cu(DDC)_2_ liposomes (8 mg/kg Cu(DDC)_2_ and 50 mg/kg lipid) using a 3 x per week for 2 weeks dosing schedule. Mean tumour volume was determined on day 18 (the humane endpoint for vehicle treated animals). Data represents the mean ± SEM (*n* = 7). * indicates statistical difference (*p* < 0.05)

Cu(DDC)_2_ has represented a formulation challenge because of its poor aqueous solubility. Previously we developed a strategy where the Cu(DDC)_2_ was synthesized inside a liposomal formulation [12]. Here we used this formulation (see Methods) to assess the activity of the Cu(DDC)_2_ in a Pt-resistant A2780-CP subcutaneous tumour xenograft model. The data, shown in Fig. 4, indicate that Cu(DDC)_2_ engendered a statistically (*p* < 0.05) significant ~50% reduction in tumour burden when compared to the activity of the vehicle control. These data provide proof-of-concept results suggesting that liposomal copper complexes have the potential to be developed as a novel class of therapeutic agents for use in the treatment of Pt-resistant cancers.

## Discussion

Pt drugs are standard first-line therapies for the treatment of lung, ovarian, and head and neck cancers [25, 26]. While some cancers are inherently resistant, the widespread use of Pt-based therapies has resulted in the development of resistant cancers, often in the relapse setting where prognosis is poor [6, 25]. In vitro*,* cell lines can be developed to replicate this effect (e.g. A2780-CP), wherein cells are treated with low dose Pt drugs such as CDDP over extended periods of time until resistance is observed through changes in IC_50_ values [27, 28]. The two major mechanisms responsible for Pt resistance are DNA repair and Pt drug transport into cancer cells [2, 29]. Through extensive characterization, it was determined that at equivalent levels of drug accumulation, the A2780-CP cell line is 2-fold more efficient at repairing Cisplatin-DNA lesions when compared to the parental, Pt-sensitive cell line (A2780-S) [29]. The copper transporter CTR1 is the major influx transporter for cisplatin both in vitro and in vivo and has been reported to be reduced in cisplatin resistant cancers [25, 30]. This mechanism was attributed to cisplatin influx resulting in the rapid degradation of CTR1 [31]. Identifying new therapeutic entities that are active in Pt resistant cancers is a priority to improve treatment outcome for patients.

The ovarian cancer cell line A2780-CP had a clear resistance to CDDP and CBDCA when compared to the activity of these drugs in A2780-S cells. In order to test the utility of copper based therapies in platinum-resistant cancer cells, preliminary studies investigated Cu(DDC)_2_ in Pt sensitive and insensitive cell lines. Cu(DDC)_2_ was active irrespective of Pt sensitivity at equivalent doses. This Pt sensitivity-independent activity could be explained by the Cu(DDC)_2_ mechanism of toxicity which overcomes resistance mechanisms commonly seen in Pt-resistant cancers. Cu(DDC)_2_ is able to accumulate in cancer cells irrespective of CTR1 expression [32] and cytotoxicity is mediated through proteosome inhibition [16]. These results suggest that CBTs use mechanisms of cytotoxicity outside of those used by platinums and warrant further investigations in resistant cancers.

Five copper binding ligands (DDC, Pyr, Plum, 8-HQ and CQ) were tested in a panel of eight cancer cell lines of different origins (blood, lung, ovarian, head and neck) and differing sensitivity to Pt-based drugs exemplified by CDDP/CBDCA activity. Our in vitro data suggest that the cytotoxicity of ligands DDC, Pyr and 8-HQ appeared to be copper-dependent whereas the cytotoxicity of Plum and CQ were copper independent. This finding is important as both DSF (metabolized to DDC in vivo) + Cu-Gluconate and CQ were involved in clinical trials with no listed benefit [33–35]. By knowing the relationship between Cu and the activity of the complex, it can be reasoned that ligands such as DDC, Pyr and 8-HQ should be administered as copper complexes to ensure that the active (copper complexed) species reaches the cancer cells. This is supported by work performed by Katano et al. which showed that Pt resistant cancers have lower basal levels of copper, suggesting that the uncomplexed ligands would be less active [30]. In the current study, H1933 was the least sensitive to Pt as reflected by a > 10 μM IC_50_ cytotoxicity to all the ligands tested. In contrast, Plum and CQ could be used as single agents and pharmaceutical challenges associated with their solubility may be overcome using the Metaplex™ approach that we have described previously to prepare a nanomedicine formulation of Cu(DDC)_2_ with no loss in activity [12, 15]. Cu(II) dominates the coordination chemistry of copper and was the focus of this work but Cu(I) complexes with therapeutic activity do exist [9]. For example, *Han* et al. tested the Cu(I)-DDC complex in a subcutaneous tumour model of adenocarcinoma with ~60% reduction in tumour burden by day 25 [36]. This research focused on Cu(II) species for two reasons. First, Cu(I) is not stable in aqueous solutions and undergoes conversion to Cu(II) [37]. The creation of the Cu(I) complexes in aqueous solutions would require characterization to ensure that the Cu(I) and not the Cu(II) complexes was being synthesized. Second, Cu(I) and Cu(II) preferentially coordinate with different atoms. Cu(II) is able to complex “hard” donors such as nitrogen and oxygen, whereas Cu(I) is known to coordinate with thiols and thioesters [38].

Our data support the utility of copper-based therapeutics in the treatment of Pt-insensitive cancer. To date, not a single copper-based therapeutic has received FDA approval. This is in part due to the solubility challenges associated with these complexes. All copper complexes studied as well as the ligands Plum, 8-HQ and CQ are water insoluble (<0.1 mg/mL); in contrast, DDC and Pyr are water soluble as ligands but their therapeutic activity is significantly enhanced in the presence of Cu. All copper complexes had to be dissolved in DMSO (final concentration 0.5%) to allow for in vitro testing. The ICH guidelines for residual solvents in a product indicate that DMSO cannot be included at concentrations above 0.5% which would be needed to administer the complexes at relevant doses [39]. While the ligands used in this work are approved agents, the copper complexes themselves have never been administered as therapeutic agents. Thus, in vivo testing of selected copper complexes should be done in conjunction with cytotoxicity studies in healthy human cells. This was previously done for Cu(DDC)_2_ and both ligand as well as the copper complex exhibited IC_50_ values greater than 10 μM when added to primary (normal) human bronchial epithelial cells [12].

We have previously disclosed the Metaplex™ technology as a platform approach to formulate copper complexes inside liposomes in order to overcome solubility issues and to allow for parenteral administration of copper complexed ligands [12]. Thus, as a proof-of-concept we tested a liposomal formulation of Cu(DDC)_2_ in a xenograft tumour model of A2780-CP Pt-resistant ovarian cancer. In this study, we showed that liposomal Cu(DDC)_2_ (8 mg/kg) but not Cu-liposomes produced a statistically significant ~50% reduction in tumour burden when compared to the vehicle control. This difference is not considered clinically relevant (based on RECIST criteria [40]) but the results does suggest that copper complexes are a class of therapeutic that should be further investigated. Cu(DDC)_2_ pharmacokinetics for the liposomal product show rapid release from the liposome and degradation in the plasma, and as such would require further development to improve circulation lifetime and stability [12]. Also the use of this formulated Cu(DDC)_2_ preparation in combination with other agents typically combined with platinums (eg. the taxanes [41]) is warranted. The other complexes discussed are proposed as future developments to identify those which have activity as single agents in Pt resistant cancers and as those which can augment therapy in combination with traditional chemotherapeutics.

## Conclusion

Pt drugs have been widely successful in the treatment of cancer. The treatment of Pt-resistant cancers has been a pharmaceutical focus that requires new therapeutics with mechanisms of action differing from those invoked by Pt-based therapies. Copper-based therapeutics represents a new class of drugs that can address many of the challenges associated with Pt-resistant cancers. Our data show that four out of five screened ligands that form copper complexes reached IC_50_ values below 10 μM in the eight cancer cell lines regardless of their Pt sensitivity. Additionally, we tested one of these complexes (Cu(DDC)_2_) in vivo in a Pt-resistant ovarian cancer xenograft model and attained a 50% reduction in tumor volume when compared to vehicle-treated control mice. These data not only provide in vivo validation that copper-based therapeutics could be used to treat Pt-resistant cancer but also provides proof-of-concept that the Metaplex™ technology could be used to develop formulations of inherently insoluble copper complexes for pre-clinical and clinical evaluations.
